# Deciphering the Complexity of FSHD: A Multimodal Approach as a Model for Rare Disorders

**DOI:** 10.3390/ijms252010949

**Published:** 2024-10-11

**Authors:** Domenica Megalizzi, Giulia Trastulli, Luca Colantoni, Emma Proietti Piorgo, Guido Primiano, Cristina Sancricca, Carlo Caltagirone, Raffaella Cascella, Claudia Strafella, Emiliano Giardina

**Affiliations:** 1 Genomic Medicine Laboratory UILDM, IRCCS Fondazione Santa Lucia, Via Ardeatina 306-354, 00179 Rome, Italy; domenica.megalizzi.96@gmail.com (D.M.); giulia.trastulli95@gmail.com (G.T.); l.colantoni@hsantalucia.it (L.C.); emmaproiettipiorgo@gmail.com (E.P.P.); raffaella.cascella@gmail.com (R.C.); claudia.strafella@gmail.com (C.S.); 2 Department of Biomedicine and Prevention, Tor Vergata University of Rome, Via Montpellier 1, 00133 Rome, Italy; 3 Department of System Medicine, Tor Vergata University of Rome, Via Montpellier 1, 00133 Rome, Italy; 4 Neurophysiopathology Unit, Fondazione Policlinico Universitario Agostino Gemelli IRCCS, Largo Agostino Gemelli 8, 00168 Rome, Italy; guido.primiano@gmail.com (G.P.); cristina.sancricca@fondazioneuildmlazio.org (C.S.); 5 Department of Clinical and Behavioral Neurology, IRCCS Fondazione Santa Lucia, Via Ardeatina 306-354, 00179 Rome, Italy; c.caltagirone@hsantalucia.it; 6 Department of Chemical-Toxicological and Pharmacological Evaluation of Drugs, Catholic University Our Lady of Good Counsel, 1000 Tirana, Albania

**Keywords:** rare diseases, neuromuscular disorders, FSHD, multidisciplinary approach, genotype–phenotype correlation

## Abstract

Rare diseases are heterogeneous diseases characterized by various symptoms and signs. Due to the low prevalence of such conditions (less than 1 in 2000 people), medical expertise is limited, knowledge is poor and patients’ care provided by medical centers is inadequate. An accurate diagnosis is frequently challenging and ongoing research is also insufficient, thus complicating the understanding of the natural progression of the rarest disorders. This review aims at presenting the multimodal approach supported by the integration of multiple analyses and disciplines as a valuable solution to clarify complex genotype–phenotype correlations and promote an in-depth examination of rare disorders. Taking into account the literature from large-scale population studies and ongoing technological advancement, this review described some examples to show how a multi-skilled team can improve the complex diagnosis of rare diseases. In this regard, Facio-Scapulo-Humeral muscular Dystrophy (FSHD) represents a valuable example where a multimodal approach is essential for a more accurate and precise diagnosis, as well as for enhancing the management of patients and their families. Given their heterogeneity and complexity, rare diseases call for a distinctive multidisciplinary approach to enable diagnosis and clinical follow-up.

## 1. Introduction: A Comprehensive Framework of Rare Diseases

Rare disorders are a heterogeneous group of chronic, degenerative and life-threatening conditions emerging as a global health priority [1]. The World Health Organization (WHO) defines rare diseases as conditions affecting fewer than 1 in 2000 people in any WHO region. The definition of rare disease may vary according to different demographics, regions or socio-economic contexts. For instance, certain diseases are more prevalent in specific populations due to founder mutations and might be formally rare in some countries but not others. Tay-Sachs disease represents a rare pediatric disease characterized by metabolic dysfunction caused by alterations in *HEXA* gene (15q23) coding for the Hexosaminidase-A enzyme, involved in the biogenesis of lysosomes [2]. Its deficiency leads to ganglioside accumulation and impairment of the central nervous system. Despite its higher frequency among Ashkenazi Jews, with a higher incidence in Canada and France, the carrier rate in the general population is approximately 1 in 250 with a lower frequency as well [3]. Other rare disorders include Limb-Girdle Muscular Dystrophy type 2G/R7 (LGMD2G/R7), an ultra-rare neuromuscular condition initially identified within the Brazilian population. The associated gene is *TCAP* (17q12), encoding a muscle assembly-regulating factor [4]. The consequent muscular weakness initially affects the proximal pelvic muscles and then the distal legs with calf hypertrophy. However, 119 LGMD2G/R7 cases have been globally identified, with cases reported not only in Brazil but also in China and Bulgaria due to possible founder effects in the Brazilian, Eastern European and Asian populations [5].

Furthermore, Duchenne Muscular Dystrophy (DMD) is one of the most common neuromuscular disorders in childhood with an incidence between 10.71 and 27.78 per 100,000 males [6]. The genetic X-linked defects on the *DMD* gene (Xp21.2-p21.1) result in its lack of expression [7]. The protein encoded by the *DMD* gene is a component of a molecular complex acting as a bridge between the inner cytoskeleton and the extracellular matrix. Such a structure protects the sarcolemma from the force associated with stretch and contraction [8]. The pathogenic mechanism triggering DMD is related to chronic inflammation due to sarcolemma damages and myofiber necrosis in dystrophin-deficient muscles [9].

Rare diseases encompass a broad spectrum of pathologies and pathogenetic mechanisms. Despite approximately 80% of rare diseases having a genetic basis, there are also sporadic forms of infectious diseases, such as auto-immune diseases and rare cancers [10,11]. More than 7000 rare diseases have been identified, with most lacking effective treatment. The disease-underlying causes often remain unknown. Considering the difficulty in identifying and recording patients worldwide, few relevant epidemiological studies on selected rare diseases have been published [12,13,14,15]. As an example, Molster and colleagues explored the experiences of patients with rare diseases by implementing a survey to collect their experiences concerning diagnosis, information provision at the time of diagnosis, use of health-support services and involvement in research on their condition. Interestingly, 92% of the 810 respondents to the survey had received a confirmed diagnosis. Around half (51.2%) of them waited one year or more for a diagnosis, with almost one-third (30.0%) waiting five or more years. Moreover, two-thirds of the respondents (66.2%) had consulted three or more doctors for a confirmed diagnosis [12]. A proper diagnosis is often difficult to make and the follow-up by clinical centers is also challenging, complicating the comprehension of the natural history of the rarest disorders. To this purpose, applying a multidisciplinary approach combined with multiple levels of analysis represents a valuable solution to clarify complex genotype–phenotype correlations and promote an in-depth and systematic examination of rare disorders.

## 2. The Integration of Multiple Analysis Levels to Elucidate and Identify Rare Disorders

The introduction of high-throughput techniques in the genomic fields improved the molecular characterization of rare diseases by identifying several novel genes and elucidating the primary mechanism responsible for the etiopathogenesis of such conditions. For example, Next-Generation-Sequencing (NGS) technologies have transformed the diagnosis of rare and ultra-rare Mendelian disorders, allowing diagnoses that were previously inaccessible [16]. Several NGS platforms have been developed to perform short-read and long-read sequencing analysis, depending on the clinical indication [17]. NGS is advantageous for detecting Single Nucleotide Variants (SNVs) in disease-causative or disease-candidate genes implicated in rare disorders. In addition, integrating genomic, transcriptomic and epigenomic data further enhances the understanding of rare diseases and their related mechanistic insights. However, generating a large amount of data should be supported by employing advanced and efficient data analysis techniques for managing and facilitating the interpretation of the outcomes. Advanced skills in the field of computers, software and programming allow the analysis of large data sets by implementing algorithms able to provide quick and user-friendly tools to be employed for managing clinical and molecular data. Seo and co-workers developed a streamlined and automated variant prioritization system, termed “EVIDENCE” (3billion Inc., Seoul, Republic of Korea). This pipeline allows analyzing over 100,000 variants following the ACMG (American College of Medical Genetics and Genomics) criteria and prioritizes variants according to the phenotype of each patient within a few minutes. Such a tool was able to generate “similarity scores” by comparing the clinical phenotypes associated with candidate variants to the actual patient’s phenotype. Then, the tool matches such scores to genetic disorders listed in the OMIM (Online Mendelian Inheritance in Man) database. This study considerably enhanced both the efficiency and success rate of diagnosing various genetic disease with a 42.7% diagnostic yield [18]. A robust understanding of digital technologies, computer science and data-processing can, therefore, facilitate the interaction between healthcare professionals and experts from other fields and encourage an interdisciplinary approach as a combination of multiple analysis levels.

Given their diversity and complexity, a better understanding of the genetic basis of rare diseases results in more accurate prognosis, surveillance and genetic advice, which are crucial for the care of affected families. Such a comprehensive multidisciplinary approach combined with the increasing availability of data from large-scale population genetic analyses led to the enhancement of the discovery rate of causative genes and an improved diagnosis of rare diseases. Notably, the availability of cutting-edge and more accessible technologies for identifying the molecular hallmarks underpinning a rare disease can also promote the research of new therapies.

## 3. Successful Models for Rare Diseases Management

### 3.1. Integration of Genomic–Epigenomic Data and Clinical Information

The integration of genomic and epigenomic data in light of clinical evaluation provided by specialized experts should be experienced for a proper genotype–phenotype correlation and, subsequently, for the diagnosis and follow-up of patients and their families. The diagnostic path of rare disorders starts with defining the patients’ phenotype. Data digitization and the use of advanced algorithms proved to be useful in supporting the implementation of several bioinformatic tools consulted by researchers, biologists, data scientists and professional figures working on rare conditions. Most of them are developed with the attempt to standardize data provided by clinical examinations. For example, the Human Phenotype Ontology (HPO) is a database serving as a vocabulary for describing phenotypic abnormalities occurring in more than seven thousand diseases [19]. It is considered a reference tool for deep phenotyping in rare diseases and it is internationally adopted by numerous academic and commercial organizations. In the field of translational research, HPO was used to identify new drug targets or extract peculiar phenotypes to explain the contribution of novel or candidate genes in driving molecular pathways associated with specific diseases [20,21]. As an example, Pinto and colleagues investigated rare Copy-Number Variations (CNVs) and Single Nucleotide Variants (SNVs) in 2446 families affected by autism spectrum disorder (ASD). They only selected only implicated in dominant, recessive, or X-linked disorders with neurological phenotypes on the basis of the HPO source. Such a strategy allowed prioritizing the key/candidate ASD-associated genes disrupted by CNVs and uncovering biological pathways (such as neuronal signaling and development, synapse function and chromatin regulation) common to these genes [22]. However, such a database is constantly updated by integrating genetic data from massive parallel sequencing techniques and enriching clinical information on rare diseases. The solely clinical evidence is often not enough to provide an accurate diagnosis, especially for diseases characterized by similar phenotype or overlapping clinical course. These conditions necessarily require a deeper investigation of the molecular hallmarks contributing to the disease presentation. A unifying strategy for managing rare disorders is crucial to ensure a reliable and accurate diagnosis, reduce diagnostic delay and prioritize patients requiring further molecular investigation. Da Costa and co-workers combined genome sequencing and optical genome mapping (OGM) analyses to elucidate the composition of a complex structural variant, namely 9p24 rearrangements, segregating in a family with a congenital heart defect. Overall, the study relied on integrating multiple analyses, including karyotype, chromosomal microarray analysis, Fluorescent In Situ Hybridization (FISH), genome sequencing, RNA-seq and OGM. This combined approach revealed a novel genetic biomarker to be investigated along the reproductive perspectives of affected individuals [23].

The integrative approach enhances diagnostic accuracy and the development of targeted therapies and accelerates the translation of research findings into clinical practice. Importantly, the integration of diverse data types, including genomic, transcriptomic and epigenomic information, takes advantage of advanced Artificial Intelligence (AI) approaches and contributes to providing a multimodal understanding of rare diseases.

### 3.2. The Implementation of Artificial Intelligence in the Management of Rare Diseases

The role of AI has become increasingly pivotal nowadays. The adoption of AI approaches boosted the healthcare system by improving big data analysis and developing tools to support the disease diagnosis and treatment. AI and Machine Learning (ML) algorithms represent powerful tools for handling the vast amounts of data generated by NGS and other high-throughput techniques. These technologies can identify patterns and correlations that might be missed by traditional analytical methods, thereby accelerating the discovery of disease-associated genetic variations. AI’s ability to learn from complex datasets allows for more accurate predictions and insights into the genotype–phenotype relationships in rare diseases. As an example, ML approaches have proven to be invaluable in clinical research by enabling the extraction of disease-relevant patterns from clinical and molecular data. However, the rarity of such conditions often limits the availability of the samples, posing a challenge for the effective application of ML. To address this, establishing collaborative networks among specialized centers and conducting multicentric studies can significantly increase sample size and diversity, making ML more feasible and effective.

Despite each rare disease affecting a relatively small number of individuals, the cumulative number of people affected by all the rare diseases is substantial. This aspect emphasizes the importance of generating high-dimensional datasets, which are essential for the successful application of ML-based analyses. High-dimensional data allows ML algorithms to identify subtle patterns and correlations that might go unnoticed, leading to deeper insights into the etiology and progression of rare diseases. Moreover, AI can incorporate multi-omics data (genomic, transcriptomic and epigenomic) to provide a comprehensive view of the molecular base underpinnings of rare diseases, leading to more precise diagnostic and therapeutic strategies. Genetic variants in noncoding regions detected by the analysis of the transcriptome and the genome may be responsible for a noteworthy percentage of causal mutations in rare genetic conditions [24,25]. Jaganathan and colleagues employed a neural network architecture consisting of 32 dilated convolutional layers to implement an advanced bioinformatic tool for the prediction of the pre-mRNA transcript position (splice donor, splice acceptor or neither). This deep learning approach enables the recognition of sequence determinants spanning very large genomic distances. This strategy provides a solution to the possible presence of tens of thousands of nucleotides separating the splice donors and splice acceptors sites. In fact, the neural network learned splicing determinants directly from the primary nucleotide sequence by evaluating 10,000 nucleotides from the flanking context sequence to precisely predict the splice function of each position in the pre-mRNA transcript.

These findings were crucial considering that genetic alterations in noncoding regions with predicted splice-altering consequence are strongly deleterious in the human population [26].

### 3.3. The Cooperation and Sharing of Data among Different Institutes

As already mentioned, the involvement of multiple centers when recruiting patients for a research study on a rare disease overcomes the challenge of small sample sizes generally arising in the case of less common disease conditions. The accessibility to a larger amount of data collected from diverse institutes enhances the statistical power of the study, making results more reliable and robust. Moreover, multi-center studies collaborations foster the development of standardized protocols and methodologies, essential for comparing outcomes and making the data more actionable.

Amyotrophic Lateral Sclerosis (ALS) and Frontotemporal dementia (FTD) are neurodegenerative disorders with shared pathogenic mechanisms and genetic signatures. Several studies reported that the hexanucleotide expansion of a non-coding region within the *C9orf72* gene (9p21.2) leads to the development of ALS and/or FTD with variable clinical expression and age-dependent penetrance [27]. Although being recommended for patients with a positive family history of ALS, FTD, or both, the genotyping of the *C9orf72* hexanucleotide repeat expansion has been useful for the revaluation of sporadic ALS and FTD patients. Such extensive genotyping pointed out the presence of the hexanucleotide repeat expansion in *C9orf72* in many cases, thus suggesting the almost certain existence of still-unrecognized affected cases. Moreover, about 35% of *C9orf72* patients have an atypical presentation similar to other neurodegenerative disorders (Parkinson’s disease, Huntington’s disease, Lewy body dementia, Alzheimer’s disease and parkinsonism) that can lead to misdiagnosis [28]. The recognition of *C9orf72*-associated phenotypes is therefore challenging. The absence of a defined cutoff for the hexanucleotide repeats expansion that distinguishes between healthy and affected subjects further complicates the diagnosis process and the interpreting of genotypes, especially in the presence of borderline repeat numbers. In addition, the lack of a standardized protocol for assessing the *C9orf72* repeats expansion raises the need to harmonize *C9orf72* testing and define a cutoff. Giardina and colleagues performed a multicentric survey on a healthy Italian population with the aim of identifying the alleles associated with a healthy phenotype to ultimately support and facilitate the clinical interpretation of results. By employing standardized protocols validated by in parallel and blind proficiency testing, they collected data from 967 subjects. In particular, the study relied on the participation of 13 institutes, members of the RIN (IRCCS Network of Neuroscience and Neurorehabilitation), based in Italy [29]. The construction of a network allowed sharing of the findings, specimens and approaches with scientific and medical communities nationwide and promoted a centralized approach to the disease.

Usher syndrome (USH) is the most common cause of deafness and blindness in humans, with a prevalence of about 1/10,000 [30]. Usher syndrome has three subtypes, highly heterogeneous from a genetic and clinical point of view and characterized by sensorineural hearing loss and Retinitis Pigmentosa (RP), with or without vestibular dysfunction [31]. Among the plethora of genes associated with USH subtypes, *MYO7A* (11q13.5) is one of the genes responsible for the USH1 subtype, accounting for approximately 40% of all USH cases [30,32]. Testa and colleagues performed the first prospective longitudinal analysis in one of the largest cohorts of European patients (*n* = 50) with USH1B carrying biallelic pathogenic variants in *MYO7A*. They conducted a multicentric study for improving the understanding of the natural history of the disease by describing for the first time the progression of the disease in a prospective longitudinal setting. The study confirmed the relatively slow natural course of the disease, which complicates the identification of reliable and reproducible outcomes to adopt in gene therapy trials. This multicentric study allowed for a more precise phenotyping of heterogeneous disease, which is essential for the prognosis, clinical trials planning, intervention timing and the establishment of relevant therapeutic outcome measures [33]. Therefore, the recruitment of patients from different clinical and research centers can provide insights on the disease manifestation across various populations, improving the reproducibility of the findings.

These studies thereby provide an effective model of collaboration between institutes, emphasizing the importance of sharing genomic data and standardizing analysis techniques to encourage translational research on challenging diseases.

## 4. The FSHD Diagnostic Path: An Ideal Benchmark for How to Tackle Rare Diseases Challenges

### 4.1. Focus on Facio-Scapulo-Humeral Dystrophy (FSHD)

Facio-Scapulo-Humeral muscular Dystrophy (FSHD) represents a muscular condition where the multidisciplinary approach proved to be essential to achieving a more accurate and precise diagnosis and improve the management of patients and their families. FSHD is the third most common type of muscular dystrophy with a prevalence of approximately 12/100,000 individuals [34]. Considering its heterogeneous clinical presentations and complex molecular genetic basis, the FSHD diagnosis may benefit from a multidisciplinary approach to define an accurate genotype–phenotype correlation. FSHD is known to be characterized by an autosomal dominant pattern of inheritance, although incomplete penetrance and variable expressivity among patients and within families are frequently observed. FSHD patients typically manifest asymmetric weakness affecting facial, scapular and humeral muscles, eventually involving other muscle groups such as the abdominal and lower limb muscles in later phases of disease. Different FSHD scores have been proposed to facilitate the standardization of the clinical evaluation [35,36,37]. The onset of FSHD is usually between the second and third decade of life, although clinical manifestations can also occur in the infantile or late age [38].

**Figure 1:**
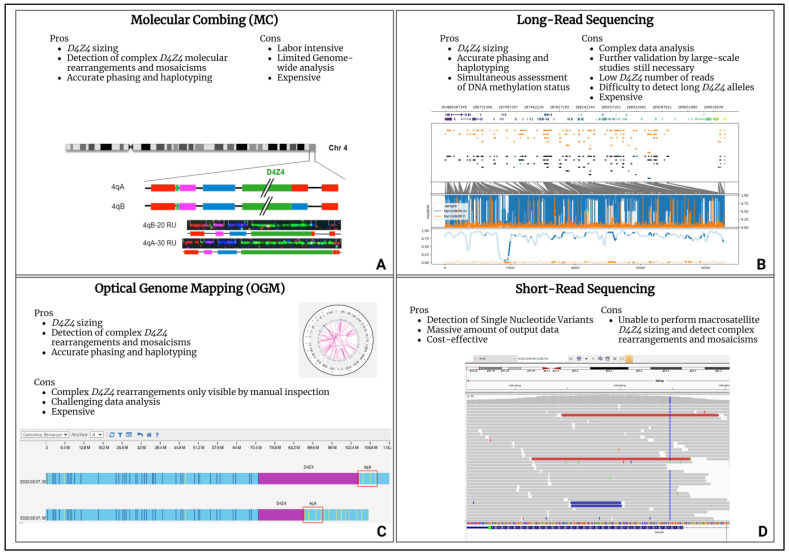
High-throughput technologies for FSHD diagnosis. (**A**) Molecular combing (MC) [48]. (**B**) Long-read sequencing. (**C**) Optical genome mapping (OGM). (**D**) Short-read sequencing. The figure reports the advantages and disadvantages of the techniques. Each panel contains an example of the data plot for the secondary analysis provided by specific software and bioinformatic pipelines.

From a molecular point of view, FSHD pathogenesis has been associated with the aberrant expression of the *DUX4* gene (4q35.2), coding for a transcription factor that is selectively expressed in cleavage stage embryos, until being epigenetically repressed in adult muscle cells [39]. A *DUX4* gene is embedded in each Repeated Unit (RU) of the *D4Z4* locus (4q35). FSHD-affected patients carry the 4qA permissive allele containing, in the most-distal *D4Z4* RU, the Poly-Adenylation Signal (PAS) allowing *DUX4*-mRNA stable transcription and translation, which is toxic for adult muscle cells [40]. Besides a permissive 4qA allele, FSHD is also characterized by the *D4Z4* epigenetic de-repression caused by two mechanisms. The first consists of the presence of a *D4Z4* with 1-10 RU (instead of the 11-150 RU of healthy subjects) and it is typical of the most recurrent form (95% of patients), known as FSHD1 (OMIM #158900). The second mechanism is associated with the presence of pathogenic variants within *D4Z4* epigenetic modifiers, such as the *SMCHD1* (18p11.32), *LRIF1* (1p13.3) and *DNMT3B* (20q11.21) genes, together with the 11-20 RU 4qA allele, and is responsible for the FSHD2 form (OMIM #158901) that affects 5% of FSHD patients [41,42,43,44]. However, compound forms of the disease with both a *D4Z4* Reduced Allele (DRA) and pathogenic variants in FSHD2 genes have been described [45,46]. Notably, DRA with a reduced penetrance have been reported, whose occurrence contributes to explaining different phenotypes among members of the same family. Moreover, the presence of reduced alleles was also detected in 3% of the general population, supporting the complexity in deciphering the molecular triggers of FSHD [47]. In the presence of a clinical suspicion of FSHD, the molecular test commonly employed for the detection of the DRA is the Pulsed-Field Gel Electrophoresis (PFGE) and Southern Blotting followed by P13-E11 probe hybridization to the *D4Z4* locus. Then, in the case of borderline/long (8-20 RU) 4qA alleles, NGS techniques are employed to investigate the presence of pathogenic variants within FSHD2 genes. The availability of updated and innovative molecular assays (molecular combing, optical genome mapping and long-read sequencing) for the comprehensive evaluation of the *D4Z4* locus allowes for explaining atypical or incomplete phenotypes in subjects displaying complex mosaicisms, *D4Z4* rearrangements, in-cis duplications and DPED alleles (Figure 1) [48,49,50].

Optical genome mapping (OGM), molecular combing (MC) and long-read NGS platforms all provide *D4Z4* sizing and 4q/10q haplotyping, although relying on different techniques. In regard to the long-read NGS approach, for example, the employment of enrichment strategies can enhance the *D4Z4*-size detection. Unlike the other above-mentioned techniques, the long-read sequencing method can also determine the *D4Z4* methylation status, thereby representing a valuable tool for a better characterization of both the genetic and epigenetic features of FSHD. Moreover, the recently developed tool for the enrichment of the target region (namely Cas-9 targeting) further improved the *D4Z4* read-depth which, in turn, allowed for a highly reliable array sizing [51]. However, these next generation cytogenomic approaches still have limitations, especially related to their costs and the need for highly specialized personnel and bioinformatic facilities for data analysis. As emerging techniques, they face several issues including the scarce availability of standardized protocols due to the restricted access to instruments and the need for validation by large-scale studies. Among the high throughput technologies to be employed in the FSHD diagnostic workflow, the short-read NGS technologies enable the fast, affordable and high-quality sequencing of FSHD2 genes. However, as regard structural variants, they might give false positive results, depending on the size and the type of the variants due to the difficulty in detecting certain regions of the genome (e.g., complex or highly repetitive regions, etc.).

Along with the assessment of FSHD genetic signatures, the exploration of the *D4Z4* epigenetic landscape provided critical insights into the disease’s molecular framework, showing a robust epigenetic component underlying FSHD and its potential application for diagnostic purposes [52]. Importantly, clinical information provided by expert neurologists at the time of the patients’ recruitment is pivotal for correctly interpreting the molecular results.

### 4.2. The Multimodal Approach for the FSHD Diagnostic Path

A multimodal approach employed in the FSHD diagnostic path proves to be an ideal model for tackling rare diseases’ challenges. In fact, the complex genotype–phenotype correlation of FSHD requires in-depth and integrated management, especially due to the complex molecular context in which the disease occurs [53]. A multidisciplinary team of professionals across various medical disciplines is crucial for providing an accurate diagnosis, considering the variable age of onset and the disease’s clinical variability and heterogeneity.

FSHD patients can display a variety of symptoms ranging from severe muscle involvement in early-onset patients to subclinical multi-system involvement or the asymptomatic status of adult forms [54]. Therefore, the employment of standardized protocols and multidisciplinary care in managing the diverse manifestations of FSHD across age groups is essential. In addition, rapid innovation in computer science has promoted an increasing interest in artificial intelligence (AI) approaches in the last few years. AI is rapidly transforming medicine by supporting the diagnosis, prognosis and assessment of therapeutic targets. The employment of ML and Deep Learning (DL) approaches to manage rare disorders has been widely described [55]. ML-based methods have been tested to enhance the usefulness of molecular disease biomarkers, highlighted by genetic, epigenetic, clinical and radiological analyses [52,56,57]. The availability of a standardized pipeline developed by applying ML algorithms can enhance diagnostic accuracy by enabling the analysis of more data than previously. For example, Monforte and co-workers used a ML approach on data provided by the radiological investigation of 295 patients. In detail, they studied 187 FSHD patients and 108 non-FSHD ones, including subjects affected by both hereditary and acquired myopathies to identify patterns of muscle involvement to be employed in distinguishing the two groups. They found a significant association between FSHD diagnosis and specific muscular impairment (trapezius involvement, bilateral subscapularis sparing, bilateral iliopsoas sparing, asymmetric involvement of upper and/or lower limb muscles) [57]. Caputo and colleagues similarly applied ML approaches to select the most critical CpG sites at *D4Z4* locus whose methylation levels could distinguish FSHD patients from non-FSHD ones. They assessed the DNA methylation of two regions (namely DR1 and *DUX4*-PAS) within *D4Z4* locus on 335 patients (160 FSHD and 175 non-FSHD). The obtained data have been employed to implement a classifier that is able to predict the presence of FSHD1/2 genetic signatures based on the well-known reduction of *D4Z4* methylation levels. Such a study achieved high sensitivity and specificity metrics. It proved the reliability of DNA methylation as an epigenetic biomarker to be integrated with genetic and clinical data for a multi-layer characterization of FSHD patients [52].

Furthermore, Katz et al. analyzed a prospective cohort from an extensive registry of FSHD patients in the USA to determine the predictors of outcomes such as the need for wheelchair use. They evaluated data from 578 FSHD1 patients by using supervised ML models. By employing a ML-based approach to different features (e.g., clinical status, outcome of genetic test, gender etc.), the authors highlighted that the presence of several medical comorbidities, including breathing problems, pneumonia, arthritis, constipation, heart problems and psychiatric concerns, was predicted to increase the risk for progression to wheelchair use. Moreover, the presence of shorter DRAs was predictive of an earlier age at onset and diagnosis and the likelihood of wheelchair use (mainly associated with the female gender) [56].

The multimodal approach has also been shown to be helpful in enhancing the management of FSHD patients from psychosocial, reproductive and counseling perspectives. As an example, Van de Geest-Buit and colleagues investigated the psychosocial outcomes derived from facial weakness and facial impairment in 138 FSHD patients. They focused on the physical, emotional and social consequences of FSHD as significant aspects to consider for adequate patient management. They observed that patients with high facial dysfunction experienced more fear of a negative evaluation and lower social functioning. Additionally, lower psychosocial outcomes were correlated with a younger age, pain, fatigue, walking difficulties and current or previous psychological support. Other studies have further supported their findings, encouraging the improvement of psychosocial outcomes by focusing on the facial muscle structure and function and psychosocial support [58,59]. Di Feo and co-workers also employed an integrated and multidisciplinary approach as a support into the genetic counseling process with families presenting a positive family history of FSHD and undergoing preconception and prenatal counseling. Their retrospective 13-year study reported the importance of conducting an integrated multidisciplinary path in managing reproductive decisions. They proposed the involvement of a multi-skilled team including a geneticist, a neurologist, a gynecologist and a biologist to discuss each case to provide the best service and the most appropriate planning for each couple [60]. Parisien et al. reported the experience of a 13-year-old female patient under the care of a comprehensive team in achieving a diagnosis complicated by a complex clinical condition. The patient showed a peculiar chronic progressive weakness in her right long finger with a vague injury after a fall on the ground, a non-specific weakness in the patient’s right shoulder with difficulty lifting her arm, scapular winging, a remote history of viral meningitis at the age of 14 weeks, etc. In her diagnostic path, evaluations by a sports medicine specialist, shoulder specialist and orthopedic spine specialist were therefore suggested. Given the FSHD clinical suspicion, the subsequent genetic counselling allowed the planning of a molecular test that confirmed the clinicians’ hypothesis [61]. This case demonstrates how implementing a multidisciplinary team can enhance the diagnosis and management of a rare genetic condition, especially for atypical presentations. Portaro and colleagues applied telemedicine for the clinical evaluation of four siblings affected by a severe form of FSHD, living in a rural area far away from the referral center for neuromuscular diseases. The patients were affected by a severe form of FSHD with symptoms including impaired cardio-respiratory conditions requiring mechanical ventilation and wheelchair use. 540 video conferences sessions spanning six months were performed, gathering the support of different specialties such as psychology, neurology, pneumology and expert technicians for device use [62]. The combination of telemedicine and the expertise of specialists from different medical areas emerged as a user-friendly and efficient tool for the home treatment and monitoring of FSHD patients. Maleki and colleagues performed a study including 58 participants (38 FSHD and 20 non-FSHD patients) and remotely monitored them for six weeks through an app installed on their smartphone. For six weeks, they collected daily activity data in the patients with FSHD and in non-FSHD controls such as the number of steps, sleep and app use. The study employed a logistic regression model to identify 15 features that were relevant to differentiate between the patients with FSHD and the non-FSHD subjects. Specifically, features such as app use, weight, the time spent at various locations, physical activity and sleep were important for differentiating between the two populations [63].

## 5. Discussion and Conclusions

A multimodal approach is essential for the management of patients with rare disorders. Patients with rare diseases face a multitude of challenges, such as delayed diagnosis, misdiagnosis, ineffective or missing treatments and insufficient monitoring tools. The usefulness of a multidisciplinary approach has been widely validated, showing that the complete evaluation of clinical, radiological and pathological aspects improves diagnosis and patient care [64,65].

The challenging definition of a genotype–phenotype correlation in patients with rare disorders raises the need for a multi-specialized team for properly managing the affected patients and their families. Such an approach is particularly required for conditions such as FSHD, where the broad spectrum of symptoms and phenotypes complicates the diagnostic path and decision-making, especially for atypical FSHD cases [66,67].

An update of the 2012 best practice guidelines on FSHD genetic diagnosis has been recently released to summarize the current awareness of the genetic features of FSHD and renew the international consensus on the molecular testing recommendations [68]. The working group, including FSHD European Trial Network members, aimed to provide a global perspective of the minimal requirements and recommendations for the genetic confirmation of FSHD1 and FSHD2. The variability of the disease onset and severity is considerable, as demonstrated by the presence of atypical cases or patients carrying FSHD genetic signatures but not conscious of muscle symptoms, who are highlighted only upon clinical investigation. The occurrence of a FSHD genetic signature without any symptoms (possibly due to the presence of a non-penetrant DRA) represents another case requiring a care follow-up. Therefore, evaluating the patient’s natural medical history and collecting data during the clinical examination is crucial. The genetic counseling is paramount to providing patients with the clinical and molecular issues related to the test, such as the attribution of the inheritance pattern, the description of the employed technologies and the possible identification of incidental findings or inconclusive results (pre-test genetic counseling). Genetic counseling is equally important after performing the test to explain the results and their clinical implications for patients and families. Both pre-test and post-test genetic counseling should be part of a multidisciplinary approach where neurologists and geneticists cooperate to select the most appropriate genetic test. This is crucial especially during the counseling of couples, when prenatal testing or pre-implantation genetic testing is required.

Moreover, adopting multilevel analyses is particularly relevant in the case of complex molecular rearrangements affecting *D4Z4* locus, in the course of the diagnostic path. In particular, this is important when patients display a FSHD phenotype but are negative to FSHD1/FSHD2 traditional testing. The availability of Next-Generation Cytogenomic approaches such as OGM and MC allow solving complex *D4Z4* configurations not detectable by Southern Blotting. Furthermore, such technologies and the employment of advanced analysis software can reveal the presence of in-cis 4qA allele duplication or *D4Z4* proximally extended deletion (DPED) [69]. DNA methylation at the *D4Z4* locus represents an additional level of analysis that is highly informative for the presence/absence of FSHD genetic signatures. The utility of exploring DNA methylation of the distal part of the *D4Z4* locus has been shown to predict the presence of a FSHD1 signature [52].

Moreover, the reduced methylation of the whole *D4Z4* locus (including both the proximal and the distal region) is also a well-known hallmark of detrimental variants in FSHD2 genes [70]. In both cases, DNA methylation is a valuable tool for prioritizing patients to be tested for FSHD1 or FSHD2 testing, reducing the costs and time required to provide an appropriate response and avoid unnecessary tests. Furthermore, the employment of high-throughput NGS technologies improved genomic analysis, allowing for the identification of novel candidate genes or providing a comprehensive evaluation of the disease molecular background [71]. Data provided by such analyses expand the knowledge of the individual genome and epigenome, paving the way for personalized and targeted treatment strategies [72,73,74].

**Figure 2:**
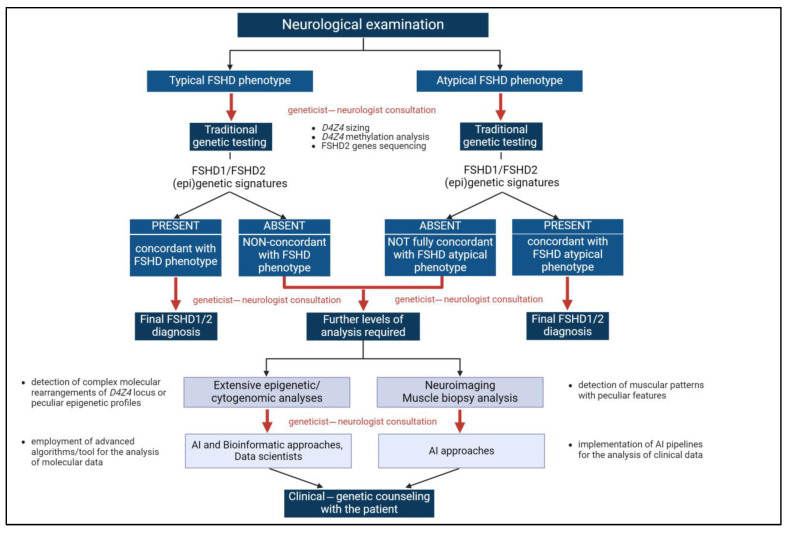
The multimodal approach for FSHD diagnosis. The integration of clinical and genetic data at different levels of the FSHD diagnostic path (corresponding to red arrows) is a pivotal component of the multimodal approach highlighted by the present work. Created with https://www.biorender.com (accessed on 24 July 2024). AI: Artificial intelligence.

During the FSHD diagnostic path, geneticists and clinicians cooperated to better accomplish the management of FSHD cases, especially peculiar conditions, by proposing deeper analyses or selecting the most appropriate interventions or therapies. This partnership helped in identifying phenotypic correlations, which may have led to recognizing disease features that might otherwise be overlooked (Figure 2).

In addition, collaborations across multiple centers gather specialists with diverse expertise and access to different technological resources. As already mentioned, such a strategy can lead to the implementation of research projects supported by a larger amount of data and adequate resources and fundings. A significant example of a recent collaboration was realized by the FSHD Italian Clinical Group. The cooperation of 18 institutes nationwide allowed for the enrollment of 218 patients to be involved in a prospective study [75]. This study validated the introduction of the protocol for evaluating the *D4Z4* methylation profile developed by Caputo and colleagues [52,76] into the traditional FSHD diagnostic path. This partnership aided the collection of enough samples for carrying out a one-year validation study [75]. Moreover, each center provided clinical information for each patient (where available) in order to enhance the understanding of genotype–phenotype correlations and explain the high inter-/intra-familiar variability. The resulting dataset contributed to enhancing the robustness of the results and provided valuable insights into the understanding of FSHD.

In conclusion, adopting an interdisciplinary approach combined with multiple levels of analysis proves to be an effective strategy for unraveling the intricate genotype–phenotype relationships that often complicate the comprehension of rare diseases. FSHD is a good example, already benefiting from this approach, which allows for achieving more accurate and precise diagnoses and, in turn, improves patient and family management. Moreover, the precise and systematic collection of clinical observations on rare genetic diseases at a national level can serve as a rich data source, further supporting the comprehensive characterization of rare disorders. From this perspective, the integration of AI in the diagnosis of rare diseases should be encouraged. AI enhances the ability to analyze complex datasets, identify patterns and predict disease progression, thus supporting more efficient and accurate diagnoses.

## Data Availability

The original contributions presented in the study are included in the article.
